# Chestnut-derived ellagitannins (FT50) protect against western diet-induced metabolic dysfunction and preserve beta cell function in mice

**DOI:** 10.3389/fendo.2026.1802808

**Published:** 2026-04-30

**Authors:** Maša Skelin Klemen, Nika Polšak, Jasmina Jakopiček, Polona Kovačič, Jan Kopecky, Eva Paradiž Leitgeb, Jasmina Kerčmar, Lidija Križančić Bombek, Jurij Dolenšek, Andraž Stožer

**Affiliations:** Institute of Physiology, Faculty of Medicine, University of Maribor, Maribor, Slovenia

**Keywords:** beta cells, calcium imaging, ellagitannins, glucose intolerance, insulin resistance, metabolic syndrome, mouse model, western diet

## Abstract

**Background:**

Western diet (WD) consumption accelerates the development of metabolic syndrome (MetS) and type 2 diabetes mellitus (T2DM). This study investigated whether chestnut-derived ellagitannins (FT50) modulate early metabolic alterations associated with WD feeding in C57BL/6J male mice.

**Methods:**

Male C57BL/6J mice were fed either a western diet (WD) or a WD supplemented with chestnut extract (FT50) for 12 weeks. Body weight, adiposity, glucose tolerance, and indices of insulin action were monitored throughout the study. Glucose and insulin homeostasis were assessed by measuring fasting glucose levels, insulin levels, and the HOMA-IR index. Pancreatic function was evaluated by *ex vivo* Ca^2+^ imaging in pancreas tissue slices to assess beta cell activation dynamics and islet network coordination.

**Results:**

WD feeding induced significant body weight gain, adiposity, hyperglycemia, glucose intolerance, changes consistent with impaired insulin action, and partially compensatory hyperinsulinemia. Supplementation with FT50 prevented weight gain and fat accumulation, reduced organ hypertrophy, and was associated with improved glucose and insulin-related parameters without affecting the caloric intake. FT50-fed mice showed improved glucose tolerance from week 4 onward, accompanied by a lower HOMA-IR values and reduced hyperinsulinemia, consistent with improved indirect markers of insulin action relative to WD-fed mice. *Ex vivo* Ca^2+^ imaging in pancreas tissue slices revealed that in FT50-fed mice, beta cells have higher activation thresholds, longer activation delays, and lower Ca^2+^ activity during the plateau phase, while their network coordination is preserved.

**Conclusions:**

In sum, these effects indicate reduced beta cell excitability and are consistent with an attenuation of early metabolic alterations under WD conditions. Overall, these findings suggest that FT50 supplementation modulates early WD-induced metabolic changes in male mice. However, the study is limited to early-stage alterations, does not address later stages of metabolic disease, and does not establish direct effects on insulin sensitivity. Within these constraints, chestnut extract represents a potentially promising bioactive dietary intervention for modulating early metabolic responses to WD.

## 1 Introduction

Metabolic syndrome (MetS) is an increasingly prevalent disorder, primarily driven by the global rise in obesity. It is strongly linked to a heightened risk of cardiovascular diseases, particularly hypertension (1) and type 2 diabetes mellitus (T2DM) (2). These risks are especially pronounced in developed countries, where MetS is emerging at younger ages, presenting a significant public health challenge (3).

The global prevalence of MetS varies significantly, ranging from 12.5% to 31.4%, depending on factors such as age, gender, race, ethnicity, and the diagnostic criteria used (4). According to the 2009 harmonized definition, metabolic syndrome (MetS) is identified by the coexistence of multiple metabolic abnormalities, such as visceral obesity, hypertriglyceridemia, hypertension, hyperglycemia, and low HDL cholesterol. A diagnosis is made when at least three of these—obesity, hyperglycemia, hypertension, or dyslipidemia—are present (5). In 2022, an updated definition was introduced, adopting a more clinically integrated approach that centers on obesity as the core criterion, accompanied by at least two of the following: elevated blood pressure, impaired glucose metabolism, or increased non-HDL cholesterol (6).

MetS represents a complex cluster of health issues with no single underlying cause. Its contributing factors can be both genetic and environmental (7). Genetic predispositions, such as a family history of T2DM, hypertension, insulin resistance, and certain ethnic backgrounds, significantly increase the risk of developing MetS (4). Additionally, modifiable environmental influences, including sedentary lifestyles, physical inactivity, and poor dietary habits, play a pivotal role in its development (8).

MetS predisposes individuals to a variety of serious health complications, including cardiovascular disease (CVD), T2DM, non-alcoholic fatty liver disease (NAFLD) (9), and non-alcoholic fatty pancreas disease (NAFPD) (10). Additionally, MetS has been linked to an increased risk of various cancers—such as liver, pancreatic, breast, and bladder cancer—as well as kidney and pancreatic dysfunction (11, 12). Managing both genetic and environmental factors is essential in preventing these adverse health outcomes, yet the precise timing and progression of metabolic changes remain uncertain.

Emerging evidence suggests that the progressive nature of T2DM is largely confined to individuals experiencing increased body mass and insulin resistance. Consequently, preventing weight gain and insulin resistance or reversing them is of paramount importance (13, 14). The most effective approach to mitigating both involves either reducing caloric intake or achieving a negative energy balance through increased energy expenditure. This necessitates a sustained energy deficit, requiring commitment, discipline, and long-term lifestyle adjustments. To ensure lasting success, the deficit should be moderate and manageable. While various over-the-counter products claim to promote short-term weight loss, none have demonstrated effectiveness in preventing weight gain, MetS, or related complications such as CVD and T2DM.

One of the most valuable methods for studying MetS and T2DM is the use of mouse models. These animal models offer numerous advantages, including genetic uniformity, controlled environmental conditions, and cost-effectiveness. Researchers employ techniques such as dietary manipulation, genetic modification, and drug administration to induce MetS in rodents (15–18). Among these, diet-based models are particularly significant, as they closely reflect the multifactorial nature of human metabolic diseases. For example, rodent strains like C57BL/6J mice exhibit a predisposition to obesity and related metabolic disorders when subjected to a high-fat (HFD) or western diet (WD), mirroring the development of MetS and T2DM in humans (19, 20). These findings underscore the importance of diet composition and rodent strain selection in advancing our understanding of metabolic disorders.

Tannins, found globally, are water-soluble polyphenolic compounds (21) that demonstrate biological properties similar to antibiotics (22, 23) and are categorized into two main groups: hydrolysable tannins (HT), composed of gallic acid and glucose, and condensed tannins (CT), derived from flavonoids (24, 25). Hardwood extracts, especially from oak and chestnut, are rich sources of HT, particularly ellagitannins. Epidemiological studies increasingly link diets rich in plant-derived bioactive compounds, antioxidants, and polyphenols to a lower risk of MetS and related conditions (26–29). At the molecular level, tannins interact with proteins via hydrogen and covalent bonds, acting as enzyme inhibitors—particularly against α-amylase, α-glucosidase, and lipase—key regulators of carbohydrate and lipid metabolism (30–35). HTs may also inhibit dipeptidyl peptidase 4 (DPP-4), offering therapeutic potential for diabetes management (36). Additionally, tannins, similarly to flavonoids, exhibit insulin-mimetic effects, enhancing glucose uptake in peripheral tissues and suppressing adipocyte differentiation, aiding in blood glucose control and anti-adipogenic actions (37–41).

Although polyphenols are metabolized in the liver, their biological effects remain incompletely understood. Their metabolites may influence pancreatic beta cell function and regulate insulin secretion, underscoring their potential metabolic impact (42, 43).

In C57BL/6J mice, prolonged feeding with a WD, high in simple carbohydrates, over 12 weeks, induces the development of MetS and a partially compensated form of T2DM (44, 45). This condition is characterized by hyperglycemia, fasting hyperinsulinemia, glucose intolerance, and insulin resistance. At this stage, pancreatic beta cells exhibit a compensatory leftward shift in glucose–insulin responsiveness, reflecting enhanced insulin secretion at lower glucose concentrations (46, 47). Similar left-shifted beta cell behavior has been reported in other early-stage diabetes and insulin-resistance models, providing a rationale for directly assessing beta cell function in this context (48–50). The objective of this study was to evaluate the potential antidiabetic effects of chestnut extract, which is rich in ellagitannins (51). If supplementing the WD with tannins can mitigate the progression of MetS and T2DM in mice, these results could significantly contribute to the development of tannin-based therapeutic formulations for human use.

## 2 Materials and methods

### 2.1 Ethical statement

We conducted the study in full compliance with national and European guidelines for the care and handling of experimental animals, following the PREPARE and ARRIVE guidelines to ensure thorough planning, reporting, and minimization of animal suffering (52, 53). The experimental protocol was approved by the Administration of the Republic of Slovenia for Food Safety, Veterinary, and Plant Protection (U34401-1/2023-6).

### 2.2 Animals

A total of 21 eight-week-old male C57Bl/6J mice (sourced from Charles River Laboratories, Germany) were used in this study. The mice were randomly assigned to groups of 3–5 by an independent investigator not involved in the conduct of the study, and housed in individually ventilated cages (IVC, Allentown, USA; bedding Mucedola Scobis Due, Italy). They were maintained under standard conditions with a 12-hour light/dark cycle (07:00-19:00-07:00), a controlled temperature of 22 ± 2 °C, and 55 ± 10% humidity. Following a 2-week quarantine period, the mice were handled three times weekly for 2 weeks to acclimate them to the experimental procedures and personnel. At 12 weeks of age, the control group was fed a Western diet (WD, D12079B, Research Diets Inc., United States) exclusively for 12 weeks. The test group, however, was fed the WD for 3 days per week (Monday morning to Thursday morning) and the WD supplemented with 30 g/kg of sweet chestnut extract (WD+FT50; Research Diets Inc., United States) for the remaining 4 days (Thursday morning to Monday morning). Details on the composition and caloric content of the WD and the WD supplemented with tannins are available in **Supplementary Table 1**. The chestnut extract was provided by Tanin Sevnica, d.d. FT50 is a standardized extract of sweet chestnut (*Castanea sativa*), characterized by a high content of ellagitannins, a subgroup of hydrolyzable tannins. The main components include vescalagin and castalagin, as well as the roburins A, B, C, D, and E. The extract contains at least 40% total ellagitannins, as determined by HPLC analysis, and at least 15% bioavailable ellagic acid quantified following acid hydrolysis. The extract was incorporated into the WD at a dose of 30 g/kg, based on previous studies demonstrating efficacy and tolerability in rodents (data not shown). A diet containing the extract was prepared by Research Diets (USA), which ensured a homogenous mixture of the extract throughout the feed. The prepared diets were stored according to the manufacturer’s instructions to maintain stability throughout the feeding period. In mice, body weight was recorded three times a week (on Mondays, Thursdays, and Fridays), and non-fasting blood glucose levels were measured weekly (on Mondays) via tail vein puncture using a handheld glucometer (OneTouch Verio Flex, LifeScan, United States). Additionally, intraperitoneal glucose and insulin tolerance tests were performed four times during the study: before initiating the diet, and after 4, 8, and 12 weeks of dietary intervention. All *in vivo* procedures, tissue collection, and outcome assessments were performed by investigators blinded to group allocation.

### 2.3 Intraperitoneal glucose tolerance test and blood insulin measurements

Food was withheld for 6 hours (typically from 07:00 to 13:00), during which animals had unrestricted access to water. This fasting duration was determined to be optimal in previous studies (54). Basal glucose levels were measured at time 0. Prior to administering the intraperitoneal (i.p.) injection, abdominal palpation was performed to check for signs of peritonitis. Following this assessment, animals received an i.p. injection of glucose (2 g/kg body weight) using a sterile 27G needle, with a total injection volume ranging from 0.1 to 0.3 mL in sterile saline. Blood glucose levels were measured at 15, 30, 60, 90, and 120 minutes post-injection. Blood samples were collected from the tail vein using the ‘tail-clip’ method, with measurements taken using a OneTouch Verio Flex glucose meter. For the initial measurement (at time 0), a small incision (<0.5 mm) was made at the tip of the tail after applying a local anesthetic (2% xylocaine gel). Gentle tail milking was used as needed to facilitate blood collection (55). Subsequent measurements involved removing the scab from the previous incision to access blood, minimizing discomfort, and allowing repeated sampling. Additionally, at time points 0, 30, and 120 minutes post-injection, approximately 50 µL of blood was drawn from the tail vein for insulin concentration analysis.

### 2.4 Insulin tolerance test

Animals were fasted for 6 hours prior to the intraperitoneal insulin tolerance test (ipITT), typically between 07:00 and 13:00, with unrestricted access to water. Basal glucose levels were measured at the -10 minute time point (prior to insulin administration), along with blood triglyceride levels (VPD, Bled, d.o.o.), as described above. Following this, animals received an i.p. injection of insulin (0.75 U/kg in saline) via a 27G needle, with an injection volume ranging from 0.1 to 0.150 mL. Ten minutes after the insulin injection, blood glucose levels were measured again. Subsequently, an i.p. injection of glucose solution (1 g/kg in saline) was administered using a 27G needle, with a volume between 0.1 and 0.3 mL. Blood glucose levels were then measured at intervals of 15, 30, 60, 90, and 120 minutes post-glucose. If plasma glucose concentration dropped below 2 mM, the animals received an additional glucose injection at a dose of 1 mg per gram of body weight. Blood glucose levels were monitored every 5 minutes thereafter until stabilization. Glucose administration 10 minutes after insulin injection was performed to prevent excessive hypoglycaemia in accordance with animal welfare considerations.

The linear rate of glucose change (K_ITT_) for each insulin tolerance test was calculated using the Equation 1:

(1) $$K_{ITT}=\frac{glc_{0}-glc_{-10}}{10}$$

where glc_0_ represents plasma glucose concentration at time 0 minutes (prior to glucose injection), and glc_-10_ is plasma glucose concentration at -10 min (prior to insulin injection).

K_ITT_ was calculated exclusively from the initial 0–10 min interval following insulin administration, i.e., prior to glucose injection. This early phase reflects the overall *in vivo* glucose-lowering response to exogenous insulin but does not represent an isolated measure of insulin sensitivity, as it is influenced by multiple physiological factors including endogenous insulin secretion, hepatic glucose output, and neuroendocrine responses. Accordingly, K_ITT_ is interpreted here as an indirect indicator of systemic insulin responsiveness rather than a direct measure of insulin action.

### 2.5 Assessment of insulin resistance and beta cell function

To evaluate insulin resistance and pancreatic beta cell function, fasting blood glucose (mmol/L) and insulin (μU/mL) levels were measured at baseline and after 4, 8, and 12 weeks of dietary intervention.

#### 2.5.1 HOMA-IR calculation

Insulin resistance was estimated using a modified Homeostatic Model Assessment of Insulin Resistance (HOMA-IR). Glucose levels were measured in millimoles per liter (mmol/L), and insulin levels were measured in microunits per milliliter (μU/mL). As the original HOMA model was developed to reflect human glucose-insulin physiology using empirically derived constants, we adapted the model to better reflect mouse physiology. Instead of the standard constant (22.5), a study-specific normalization factor (F) was used to calibrate the HOMA-IR values such that the average HOMA-IR of control animals at baseline equaled 100, as described previously (56). The modified HOMA-IR was calculated with Equation 2:

(2) $$HOMA-IR=\frac{glu\cos e\;[\frac{mmol}{L}]*Insulin[\frac{\mu U}{mL}]}{F}$$

where F is a normalization factor derived from the mean product of fasting glucose and insulin in the control group at baseline. This normalization allowed for relative comparisons of insulin resistance across time points and treatment groups within the experimental context.

#### 2.5.2 Insulin sensitivity calculation (%S)

Whole-body insulin sensitivity was calculated using the Equation 3 as the inverse of HOMA-IR, using the same normalization factor F:

(3) $$\%S=\frac{F\;}{glu\cos e\;[\frac{mmol}{L}]*Insulin[\frac{\mu U}{mL}]}\;\times 100$$

This approach ensured that the median insulin sensitivity of control animals at baseline was set to 100%, allowing for a direct comparison of insulin sensitivity changes over time and between groups.

#### 2.5.3 Beta cell function

Pancreatic beta cell function (%B) was estimated using a modified, empirically derived index based on fasting insulin and fasting glucose concentrations, assuming a linear dependence of insulin secretion on glucose. The formula was calibrated so that the median beta cell function [%] of control animals at baseline equaled 100%. The calculation was based on empirically derived coefficients using the Equation 4:

(4) $$B[\%]=\frac{\frac{1}{A\;}\times Insulin[\frac{\mu U}{mL}]}{glu\cos e\;[\frac{mmol}{L}]-\frac{B\;}{A}}\times \;100=\frac{Insulin[\frac{\mu U}{mL}]}{1,52\;\times \;(glu\cos e\;[\frac{mmol}{L}]-3,21)}\;\times 100$$

This formulation reflects the compensatory capacity of beta cells in response to insulin resistance, allowing for comparative analysis across various experimental conditions.

### 2.6 Animal sacrifice and tissue harvesting

Following 12 weeks on the diet, mice were sacrificed through exposure to high concentrations of CO_2_, followed by cervical dislocation, as outlined in Annex IV of Directive 2010/63/EU (57). Immediately after the sacrifice, blood samples were collected to measure glucose and insulin levels. Acute pancreas tissue slices were prepared for calcium imaging, while a portion of the pancreatic tissue was fixed for histological analysis and transmission electron microscopy (TEM) to evaluate ultrastructural changes. Various tissues, including the tibia, heart, lungs, kidneys, spleen, gastrocnemius muscle, soleus muscle, liver, subcutaneous fat, visceral fat, brown fat, and intestine, were harvested, evaluated for mass/size, and frozen in liquid nitrogen for further analysis. Additionally, intestinal contents were collected for subsequent laboratory analyses.

### 2.7 Pancreas tissue slice preparation

After the sacrifice, a laparotomy was performed to access the abdominal cavity. The common bile duct was distally clamped at the major duodenal papilla. Low-melting-point 1.9% agarose (Lonza), dissolved in extracellular solution (ECS) at 40 °C, was injected proximally into the common bile duct. The ECS consisted of (in mM): 125 NaCl, 26 NaHCO_3_, 6 glucose, 6 lactic acid, 3 myo-inositol, 2.5 KCl, 2 Na-pyruvate, 2 CaCl_2_, 1.25 NaH_2_PO_4_, 1 MgCl_2_, and 0.5 ascorbic acid. Following injection, the pancreas infused with agarose was cooled with ice-cold ECS and extracted. Pancreatic tissue slices (140 µm thick) were prepared using a vibratome (VT 1000 S, Leica) and collected in HEPES-buffered saline (HBS) at room temperature. The HBS composition was (in mM): 150 NaCl, 10 HEPES, 6 glucose, 5 KCl, 2 CaCl_2_, and 1 MgCl_2_, adjusted to pH 7.4 with 1 M NaOH. For calcium imaging, slices were incubated for 50 minutes at room temperature in a dye-loading solution containing 6 mM Calbryte 520 AM (ATT Bioquest), 0.03% Pluronic F-127 (wt/vol), and 0.12% dimethyl sulfoxide (vol/vol) dissolved in HBS. Unless otherwise indicated, all chemicals were sourced from Sigma-Aldrich (St. Louis, MO).

### 2.8 Calcium imaging

Calcium imaging analyses were performed on pancreatic tissue slices obtained from all mice included in the study. Individual tissue slices were transferred to a perifusion system containing 6 mM glucose in carbogenated extracellular solution (ECS) maintained at 37 °C. Each slice was exposed to a single square pulse-like stimulation using varying glucose concentrations and durations: 8 mM for 30 minutes, 10 mM for 20 minutes, or 12 mM for 15 minutes. After stimulation, slices were incubated in a solution with a substimulatory glucose concentration of 6 mM for 15 minutes, followed by stimulation with 16 mM glucose until reactivation. Imaging was performed using a Leica TCS SP5 AOBS Tandem II upright confocal system equipped with a 20 HCX APO L water immersion objective (NA 1.0) and a Leica TCS SP8 Stellaris with a 16 HC PL APO water/oil immersion objective (NA 0.7). The acquisition frequency was 10 Hz, with a resolution of 256 × 256 pixels, for the entire stimulation protocol, enabling precise quantification of intracellular calcium concentration ([Ca^2+^]_IC_) oscillations. Calbryte 520 AM was excited using a 488 nm argon laser, and the emitted fluorescence was detected by a Leica HyD hybrid detector in the range of 500–700 nm (all equipment from Leica Microsystems, Germany), as previously described (58, 59).

### 2.9 Data analysis

Time-lapse videos were exported off-line from LASAF software (Leica Microsystems, Germany) as ROI average values per frame. Recordings were discarded (i) if spatial drift during calcium imaging displaced focal plane such that identity of cells could no longer be asserted or (ii) if bleaching of fluorescent signal prevented reliable detection of oscillatory activity. ROIs were identified manually to match individual beta cells (software copyright Denis Spelič) based on three specific criteria. Firstly, beta cells were distinguished by their unique activity patterns: they showed no activity during perifusion with 6 mM ECS, and exhibited a transient initial response followed by regular, synchronized oscillations during perifusion with 8, 10, or 12 mM ECS, a characteristic of beta cells (58, 59). Further analysis was conducted using custom MATLAB scripts. Time series data were corrected for bleaching using a combination of exponential and linear fit (59). The following Equation 5 was fitted to each time trace:

(5) $$y(t)=A+B\times x(t)+C\times e^{-D\times x(t)}$$

where *A,B,C,D* present fit parameters, *x(t)* fluorescence at time point *t*.

The onset of activity was manually determined based on typical transient increase in [Ca^2+^]_IC_ and noted as initial rise in [Ca^2+^]_IC_; the end of the activity was manually determined as the time of the final oscillation at its peak amplitude. For individual oscillations during the plateau activity peak, oscillations were detected semi-automatically using peakfinder function based on manual determination of peak amplitude, and the individual oscillations were determined as time points at half-maximal amplitude. Thresholds of the peak amplitudes were adjusted for each cell to account for differences in dye loading and signal-to-noise ratio. The time points were verified for each oscillation and each cell visually. Blinding of analysis was asserted by blinding stimulatory conditions to the analyst. On the binarized data, oscillation duration (temporal difference between oscillation beginning and end) and frequency (number of binarized events in the plateau phase) were calculated. Active time was calculated per cell as fraction of cumulative oscillatory durations within the plateau phase divided by duration of the plateau phase. For statistical evaluation, individual beta cells and/or islets were used as observational units depending on the parameter analyzed, reflecting the structured and heterogeneous nature of calcium imaging data. These units were not treated as independent biological replicates equivalent to animals, but rather as biologically relevant measures of cellular activity. Accordingly, the analysis is intended to describe reproducible cell- and islet-level response patterns across animals within each dietary group, while acknowledging that these observations are nested within islets and mice and should not be interpreted as increasing the number of independent animal-level replicates.

## 3 Results

We investigated whether the addition of FT50 could prevent the onset of MetS and partially compensated T2DM in WD-fed C57BL/6J male mice. The following sections outline the effects of FT50 on key physiological parameters related to the development and progression of MetS and T2DM.

### 3.1 FT50 prevents increases in body mass, triglyceride concentrations, and hyperglycemia

**Figure 1:**
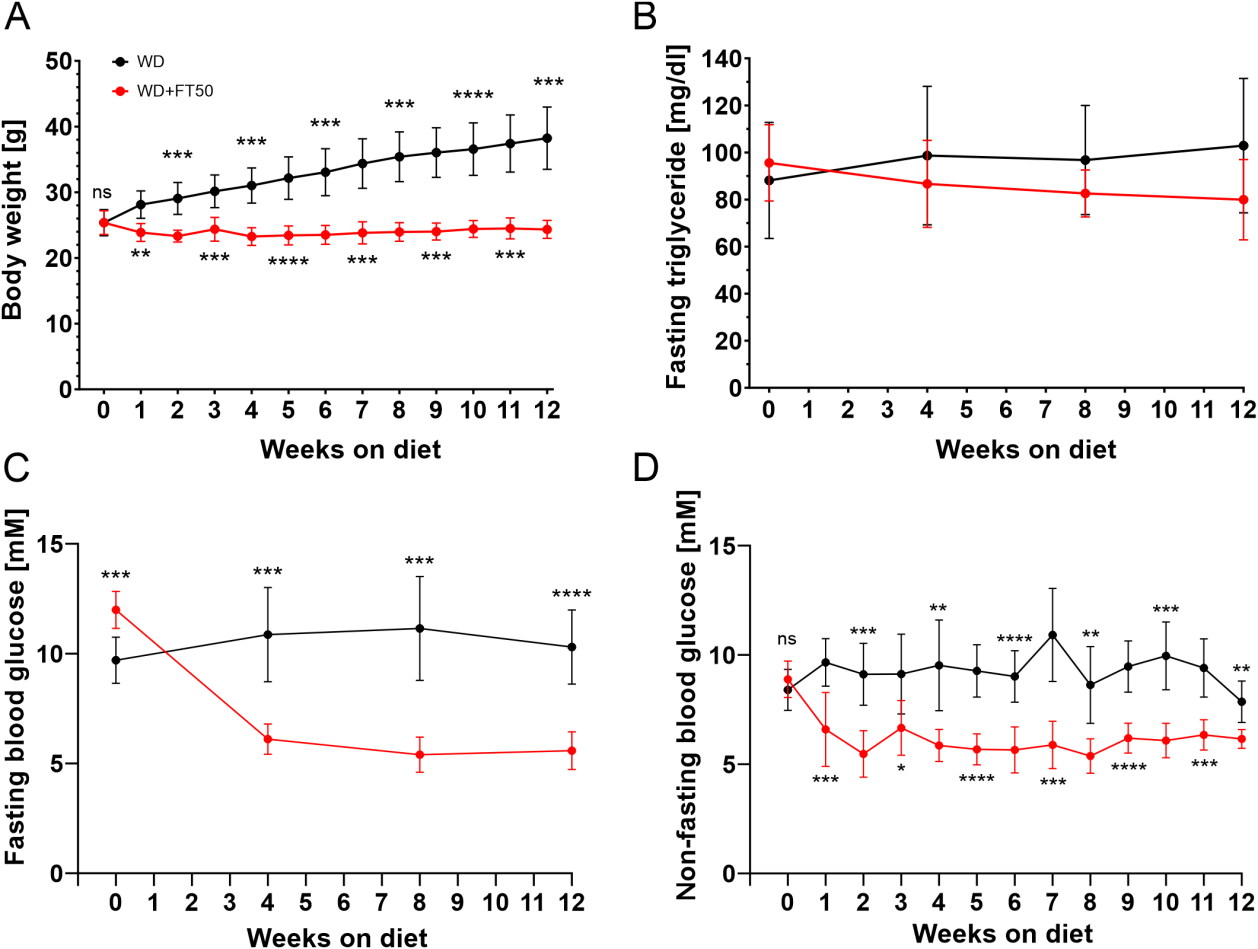
FT50 prevents diet-induced increases in body mass, triglycerides, and prevents hyperglycemia. **(A)** Body weight was significantly higher in WD mice compared with WD+FT50 (after 12 weeks: 38.3 g vs. 24.5 g, n = 9 vs. 12 mice, p = 0.0002, ***). **(B)** After 12 weeks, fasting triglyceride levels in WD+FT50 were comparable to WD (79.9 mg/dl vs. 102.9 mg/dl, n = 12 vs. 9 mice, p=0.2, ns). **(C)** Fasting glucose was decreased in WD+FT50 compared with WD (after 12 weeks: 5.6 mM vs. 10.3 mM, n = 12 vs. 9 mice, p < 0.0001, ****). **(D)** Non-fasting glucose levels were also reduced in WD+FT50 throughout the diet (after 12 weeks: 6.2 mM vs. 7.9 mM, n = 12 vs. 9 mice, p = 0.0061, **). Statistical differences between groups were assessed using 2-way ANOVA or Mixed-effects analysis followed by Šídák’s multiple comparisons test. Means ± SD are presented. * p<0.05; ** p<0.01; ***p<0.001; ****p<0.0001; ns, non significant.

After 12 weeks on the diet, mice fed the Western diet (WD) demonstrated a significant 50,7% increase in body mass (**Figure 1A**). In contrast, in animals receiving WD supplemented with FT50, body weight remained stable throughout the 12-week period, showing an overall reduction of approximately 4.1% despite consuming the same amount of calories (**Supplementary Figure 1A**). This suggests that the addition of FT50 either inhibits the reabsorption of nutrients in the gastrointestinal tract, promotes their urinary loss, increases the energy expenditure, or a combination of the above.

A striking additional observation was the marked increase in water intake in mice receiving HT, which reached nearly three times that of the WD group midway through the study (**Supplementary Figure 1B**). As the animals were not housed in metabolic cages, water consumption was measured collectively per cage, preventing statistical validation due to the limited sample size. However, the average water intake in mice on WD during 12 weeks was 0.5188 mL/g body weight, compared to 1.23 mL/g in the FT50 group. Because the increase in body weight in WD-fed animals could potentially lead to artificially low normalized values, we also calculated water intake per animal. On average, WD-fed mice consumed 16.56 mL, whereas those receiving FT50 consumed 29.52 mL (data not shown).

After 12 weeks, plasma triglyceride levels showed a tendency to decrease in the FT50 group compared to WD alone, although this difference did not reach statistical significance (p decreased from 0.9044 at baseline to 0.1954 at week 12; **Figure 1B**). In addition to fasting triglycerides, fasting glucose levels were measured four times during the diet. By week 4, fasting plasma glucose concentrations in the WD+FT50 group were significantly lower than those in the WD group (6.1 mmol in WD+FT50 vs. 10.9 mmol in the WD group, p=0.0004), and this trend persisted consistently through the end of week 12 (**Figure 1C**, 5.6 mmol in WD+FT50 vs. 10.3 mmol in the WD group, p<0,0001). Weekly non-fasting plasma glucose measurements, conducted on the same day and time each week, remained consistently lower in the WD+FT50 group compared to the WD group throughout the dietary intervention period (**Figure 1D**).

### 3.2 FT50 prevents the development of glucose intolerance

**Figure 2:**
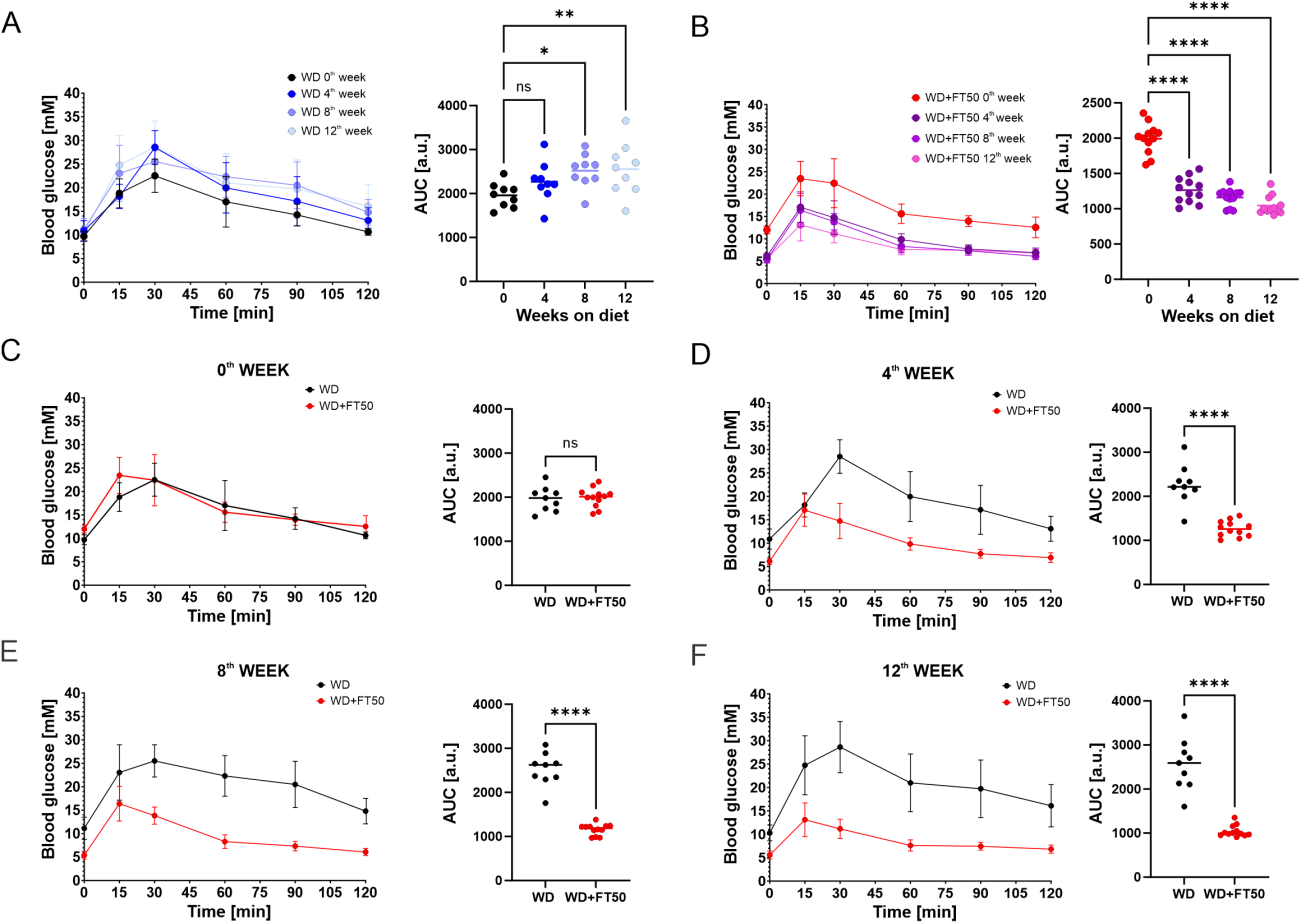
FT50 prevents the development of glucose intolerance. **(A)** Variations in glucose levels during ipGTT (left) and the area under the curve (AUC, right) for ipGTT in WD mice at 0 (WD0), 4 (WD4), 8 (WD8), and 12 (WD12) weeks. AUC increased over time (WD0: mean 1958, n = 9; WD4: 2268, n = 9; WD8: 2519, n = 9; WD12: 2556, n = 9), with significant increases from baseline at week 8 (*p = 0.0171) and week 12 (**p = 0.0047). RM one-way ANOVA with Dunnett’s multiple comparison test. **(B)** Variations in glucose levels during ipGTT (left) and AUC (right) for ipGTT in WD+FT50 mice. Compared with WD+FT50 at week 0 (mean 1993, n = 12), AUC was significantly lower at week 4 (1264, n = 12, ****p < 0.0001), week 8 (1159, n = 12, ****p < 0.0001), and week 12 (1044, n = 12, ****p < 0.0001). RM one-way ANOVA with Dunnett’s multiple comparison test. **(C–F)** Direct comparison of WD vs. WD+FT50 at 0, 4, 8, and 12 weeks. No difference at week 0 (WD: 1958, n = 9; WD+FT50: 1993, n = 12; ns). At week 4, WD AUC was higher (2268 vs. 1264, ****p < 0.0001). Differences were further enhanced at week 8 (2519 vs. 1159, ****p < 0.0001) and week 12 (2556 vs. 1044, ****p < 0.0001). Unpaired t-tests. Means ± SD are presented. ns, non significant.

Intraperitoneal glucose tolerance tests (ipGTT) were conducted prior to the diet and after 4, 8, and 12 weeks of the dietary intervention (**Figure 2**, left panels). The area under the curve (AUC) for blood glucose levels over time was calculated for each test using trapezoidal approximation, providing a summary metric of glucose tolerance (**Figure 2**, right panels). In the WD group, a significant increase in AUC was observed after 8 and 12 weeks on the diet (**Figure 2A**, right panel), indicating the development of glucose intolerance. In contrast, the addition of FT50 to the WD prevented the development of glucose intolerance and even improved glucose sensitivity, as evidenced by a significant decrease in AUC as early as 4 weeks into the diet (**Figure 2B**, left panel).

**Figure 3:**
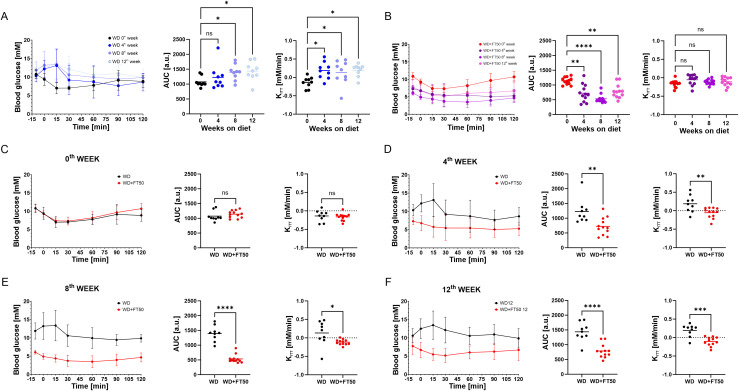
FT50 prevents the development of insulin resistance. **(A)** Comparison of glucose levels during the ipITT (left panel), area under the curve (AUC, middle panel), linear rate of blood glucose concentration change after insulin injection (K_ITT_, right) during the diet in WD mice at 0, 4, 8, and 12 weeks. AUC was progressively increased in WD-fed mice (week 0: 1073, n = 9; week 4: 1226, n = 9; week 8: 1393, n = 9; week 12: 1436, n = 9), with significant increases at week 8 (*p = 0.0338) and week 12 (*p = 0.0145) vs. week 0. K_ITT_ increased over time, showing the development of insulin resistance (week 0: –0.137, n = 9; week 4: 0.187, n = 9, *p = 0.0151; week 8: 0.132, n = 9, *p = 0.0491; week 12: 0.193, n = 9, *p = 0.0129, vs. week 0). RM one-way ANOVA with Dunnett’s multiple comparison test. **(B)** Comparison of glucose levels during the ipITT (left panel), AUC (middle panel), and K_ITT_ (right) during the diet in WD+FT50 mice at 0, 4, 8, and 12 weeks. WD+FT50 mice maintained stable insulin sensitivity over time (AUC: 1140 at week 0, 723 at week 4, 534 at week 8, 788 at week 12; all ns; K_ITT_ was also unchanged (week 0: –0.163; week 4: –0.054; week 8: –0.117; week 12: –0.107; all n = 12, ns vs. week 0).RM one-way ANOVA with Dunnett’s multiple comparison test. **(C–F)** Direct comparison of WD vs. WD+FT50 at 0, 4, 8, and 12 weeks revealed no difference in AUC at week 0 (WD: 1073, n = 9; WD+FT50: 1140, n = 12; ns), while AUC was significantly lower in the WD+FT50 group at week 4 (723 vs. 1226, **p = 0.0038), week 8 (534 vs. 1393, ****p < 0.0001), and week 12 (788 vs. 1436, ****p < 0.0001). K_ITT_ was significantly lower in WD+FT50 at week 4 (–0.054 vs. 0.187, **p = 0.0075), week 8 (–0.117 vs. 0.132, *p = 0.0201), and week 12 (–0.107 vs. 0.193, ***p = 0.0001). Unpaired t-tests. Means ± SD are presented. ns, non significant.

When comparing the data from the WD and WD+FT50 groups, no differences were observed before the diet (**Figure 2C**). However, after 4, 8, and 12 weeks on the diet, the WD+FT50 group showed a significant decrease in AUC compared to the WD group (**Figures 3D–F**, right panels). This suggests that the addition of FT50 prevents the development of glucose intolerance, a condition commonly associated with the progression of MetS and T2DM. The differences between the two groups were evident not only in the AUC values but also in the timing of peak blood glucose concentrations. In the WD+FT50 group, plasma glucose concentrations peaked 15 minutes after glucose injection, whereas in the WD group, peak plasma glucose levels were observed 30 minutes post-injection (**Figures 2D–F**, left panels).

### 3.3 FT50 prevents the development of insulin resistance

At the same time points as the ipGTT, we conducted intraperitoneal insulin tolerance tests (ipITT) to assess insulin sensitivity (**Figure 3**, left panels). We calculated the AUC for blood glucose levels over time (**Figure 3**, middle panels) and determined the rate of glucose clearing (K_ITT_) by calculating the linear slope of glucose change (**Figure 3**, right panels) to provide a supportive estimate of insulin action. A more negative K_ITT_ value indicates greater insulin responsiveness, whereas a less negative or positive K_ITT_ reflects the development of impaired insulin action.

In the WD group, a significant increase in AUC was observed after 8 and 12 weeks on the diet (**Figure 3A**, middle panel), indicating a progressive development of impaired insulin action. This was also evident from an increase in K_ITT_ as early as 4 weeks into the diet (**Figure 3A**, right panel). In contrast, the addition of FT50 to the WD attenuated these changes, as evidenced by a significant decrease in AUC (**Figure 3B**, middle panel) and unchanged K_ITT_ during the diet (**Figure 3B**, right panel).

When comparing the data from the WD and WD+FT50 groups, no differences were observed before the diet (**Figure 3C**). After four weeks on the WD, signs consistent with insulin resistance, one of the first signs of MetS and T2DM, developed. However, supplementing FT50 effectively prevented its onset, which is evident from a significantly lower K_ITT_ compared to the WD group (**Figures 3C–F**, right panels), where insulin resistance persisted throughout the duration of the diet. In contrast, mice in the WD+FT50 group maintained stable insulin responsiveness until the end of the study.

### 3.4 FT50 supplementation attenuates hyperinsulinemia observed in WD

To evaluate the effect of FT50 tannin supplementation on glucose-induced insulin secretion, plasma insulin levels were measured at baseline (0’), 30 minutes (30’), and 120 minutes (120’) during intraperitoneal glucose tolerance tests (ipGTT) conducted at baseline and after 4, 8, and 12 weeks of dietary intervention in WD and WD+FT50 groups.

**Figure 4:**
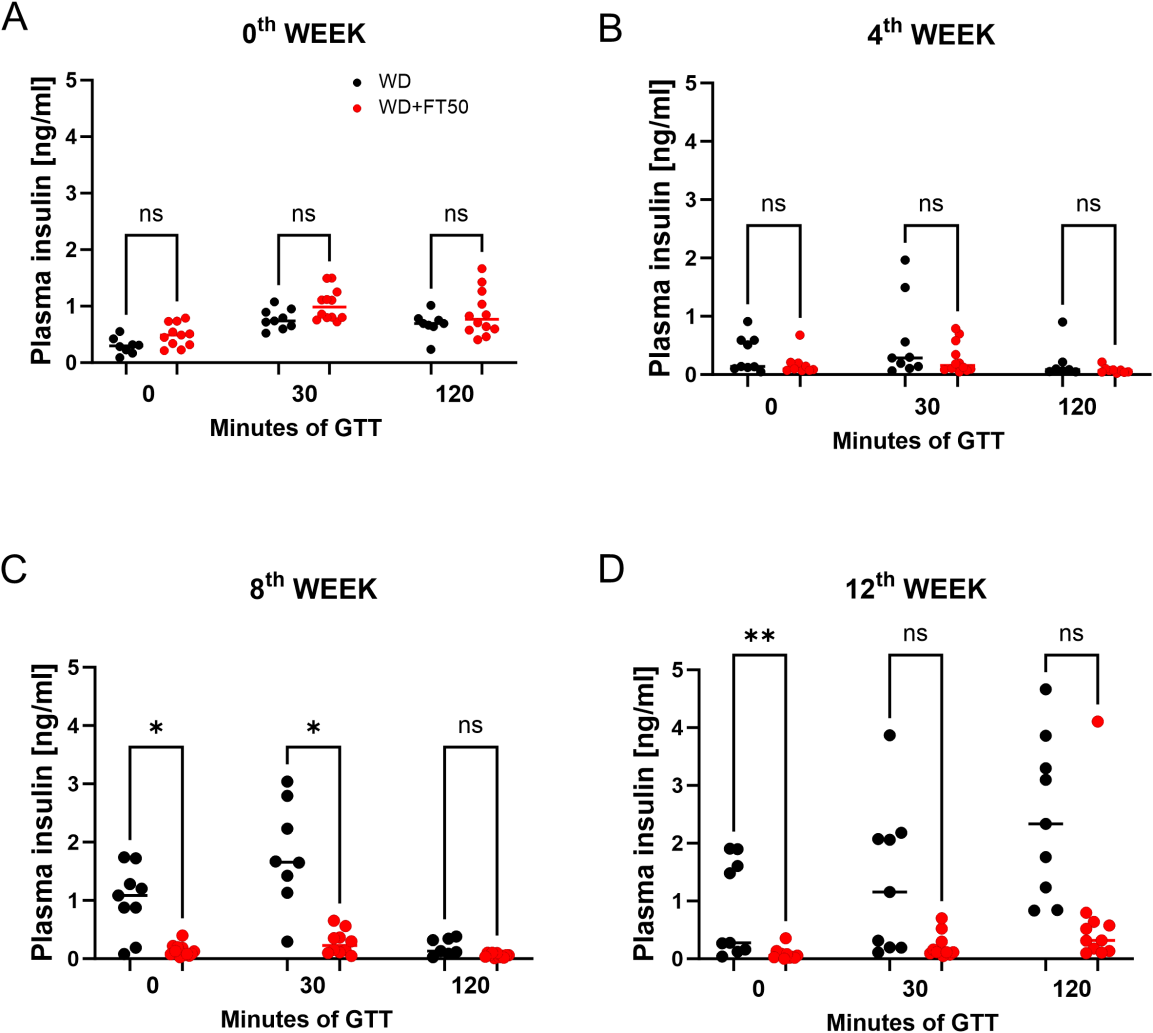
FT50 lowers plasma insulin levels during ipGTT in WD-fed mice. Plasma insulin concentrations (ng/dL) were measured at baseline (0’), 30 minutes (30’), and 120 minutes (120’) following intraperitoneal glucose injection in mice fed a WD or WD+FT50. **(A)** At week 0, plasma insulin levels did not differ between WD (0 min: median 8.125, n = 8; WD+FT50: 18.18, n = 11, p = 0.6457; 30 min: WD 35.33, n = 9; WD+FT50 47.67, n = 12, p = 0.3278; 120 min: WD 29.69, n = 8; WD+FT50 36.46, n = 12, p > 0.9999). **(B)** At week 4, no significant differences were observed between WD (0 min: 34.67, n = 9; 30 min: 37.72, n = 9; 120 min: 22.07, n = 7) and WD+FT50 (0 min: 26.78, n = 9, p = 0.8886; 30 min: 31.04, n = 12, p > 0.9999; 120 min: 13.39, n = 9, p = 0.8465). **(C)** At week 8, insulin levels were reduced in WD+FT50 compared with WD (0 min: WD 42.89, n = 9; WD+FT50 21.67, n = 12, p = 0.0112; 30 min: WD 50.25, n = 8; WD+FT50 30.40, n = 10, p = 0.0351; 120 min: WD 26.14, n = 7; WD+FT50 10.73, n = 11, p = 0.1642). **(D)** At week 12, insulin levels were lower in WD+FT50 compared with WD at 0 min (WD 33.56, n = 9; WD+FT50 10.18, n = 11, p = 0.0087), while differences at 30 min (WD 40.22, n = 9; WD+FT50 21.64, n = 11, p = 0.0537) and 120 min (WD 51.11, n = 9; WD+FT50 32.36, n = 11, p = 0.0508) did not reach statistical significance. One-way ANOVA and Dunn’s multiple comparison tests. Means ± SD are presented. ns, non significant; * p<0.05; ** p<0.01.

At baseline (week 0), no significant differences in plasma insulin levels were observed between the two groups at any time point, indicating comparable insulin responses prior to the dietary intervention (**Figure 4A**). After 4 weeks on the diets, insulin levels remained low and similar in both groups across all time points, with no significant changes detected (**Figure 4B**). By week 8, the WD group exhibited hyperinsulinemia, whereas the WD+FT50 group showed significantly lower insulin levels at 0 and 30 minutes (*p < 0.05), while levels at 120 minutes were unchanged (**Figure 4C**). These results suggest that FT50 supplementation reduces basal and early-phase insulin secretion, in line with an improvement in insulin sensitivity. At week 12, this effect was more pronounced, with a highly significant reduction in fasting insulin (**p < 0.01) in the WD+FT50 group (**Figure 4D**). Although differences at 30 and 120 minutes did not reach statistical significance (p=0.0537 and 0.0508, respectively), insulin levels remained consistently lower across all time points in the FT50-treated group, indicating a sustained trend toward enhanced insulin sensitivity.

Overall, these findings suggest that insulin secretion progressively increased in the WD group in parallel with weight gain and worsening glucose tolerance, reflecting a compensatory hyperinsulinemic response. In contrast, FT50 supplementation attenuated this rise in insulin levels—particularly during fasting and early post-glucose challenge phases—highlighting its potential role in improving insulin sensitivity over time.

### 3.5 Effects of FT50 on insulin resistance, sensitivity, and beta cell function

To assess the impact of FT50 on pancreatic beta cell function, fasting glucose and insulin levels were measured in both groups during the performed ipGTT. These values were used to calculate insulin resistance (HOMA-IR), insulin sensitivity (%S), and beta cell function using modified formulas as described in the Methods. HOMA-IR is a widely used method for estimating insulin sensitivity in mice, analogous to its application in humans, although it represents an indirect measure.

**Figure 5:**
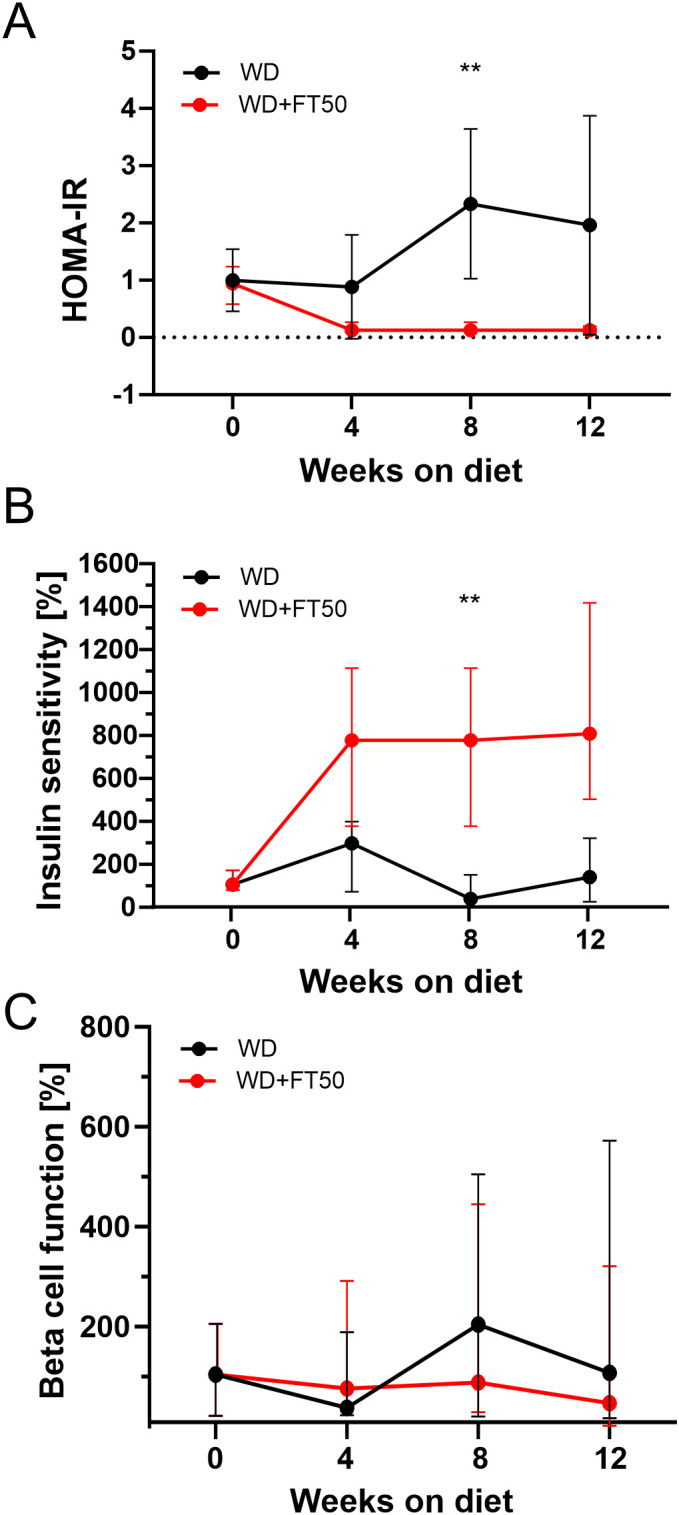
Effects of FT50 supplementation on insulin resistance, insulin sensitivity, and beta cell function in WD–fed animals. Fasting glucose and insulin were measured at baseline and after 4, 8, and 12 weeks of dietary intervention. From these values, insulin resistance (HOMA-IR), insulin sensitivity (%S), and beta cell function (%) were calculated using modified formulas as described in the Methods. **(A)** HOMA-IR was significantly lower in WD+FT50 compared with WD at week 8 (means: 0.63 vs. 2.08; n = 12 vs. 9; Šídák’s test: p = 0.0050, **) and showed a tendency toward reduction at week 12 (0.20 vs. 2.00; n = 12 vs. 9; p = 0.0861). **(B)** Insulin sensitivity (%S) was significantly higher in WD+FT50 compared with WD at week 8 (617 vs. 59; n = 12 vs. 9; p = 0.0063, **) and showed a tendency toward an increase at week 12 (750 vs. 150; n = 12 vs. 9; p = 0.0628). **(C)** Beta cell function (%B) did not differ significantly between groups but showed a tendency toward reduction in WD+FT50 compared with WD at week 12 (mean 63 vs. 214; n = 12 vs. 9; p = 0.0769). Mixed-effect analysis and Šídák’s multiple comparison tests. Means ± SD are presented.

Markers of insulin resistance and sensitivity were primarily derived from fasting glucose and insulin levels and corresponding HOMA-IR calculations. Insulin resistance in WD-fed mice was confirmed using both ipITT, by including K_ITT_ calculations of glucose disappearance rate (**Figure 3**), and HOMA-IR derived from fasting glucose and insulin levels (**Figure 5A**). However, given the modified ITT protocol, K_ITT_ should be interpreted with caution as an integrated *in vivo* response rather than a direct measure of insulin sensitivity, while HOMA-IR and fasting parameters represent the primary indicators. Mixed-effects modeling revealed significant differences in HOMA-IR between groups at week 8, with WD-fed mice showing higher HOMA-IR than WD+FT50 mice, consistent with increased insulin resistance, indicating that FT50 supplementation prevented the exacerbation of insulin resistance. Consistently, insulin sensitivity (%S), calculated as the inverse of HOMA-IR, did not change in WD-fed mice, whereas it remained significantly higher in the WD+FT50 group at week 8 (**Figure 5B**), consistent with improved insulin sensitivity relative to WD-fed controls.

Beta cell function was highly variable and did not differ significantly between groups (**Figure 5C**). Thus, FT50 was associated with improved markers of insulin resistance and preserved insulin sensitivity based on indirect indices, without significantly altering basal beta cell function within the timeframe studied.

Overall, these results demonstrate that FT50 supplementation attenuates markers of WD-induced insulin resistance and is consistent with improved insulin sensitivity, while acknowledging that these conclusions are based on indirect measures, while leaving beta cell function largely unchanged.

### 3.6 FT50 decreases fat accumulation

**Figure 6:**
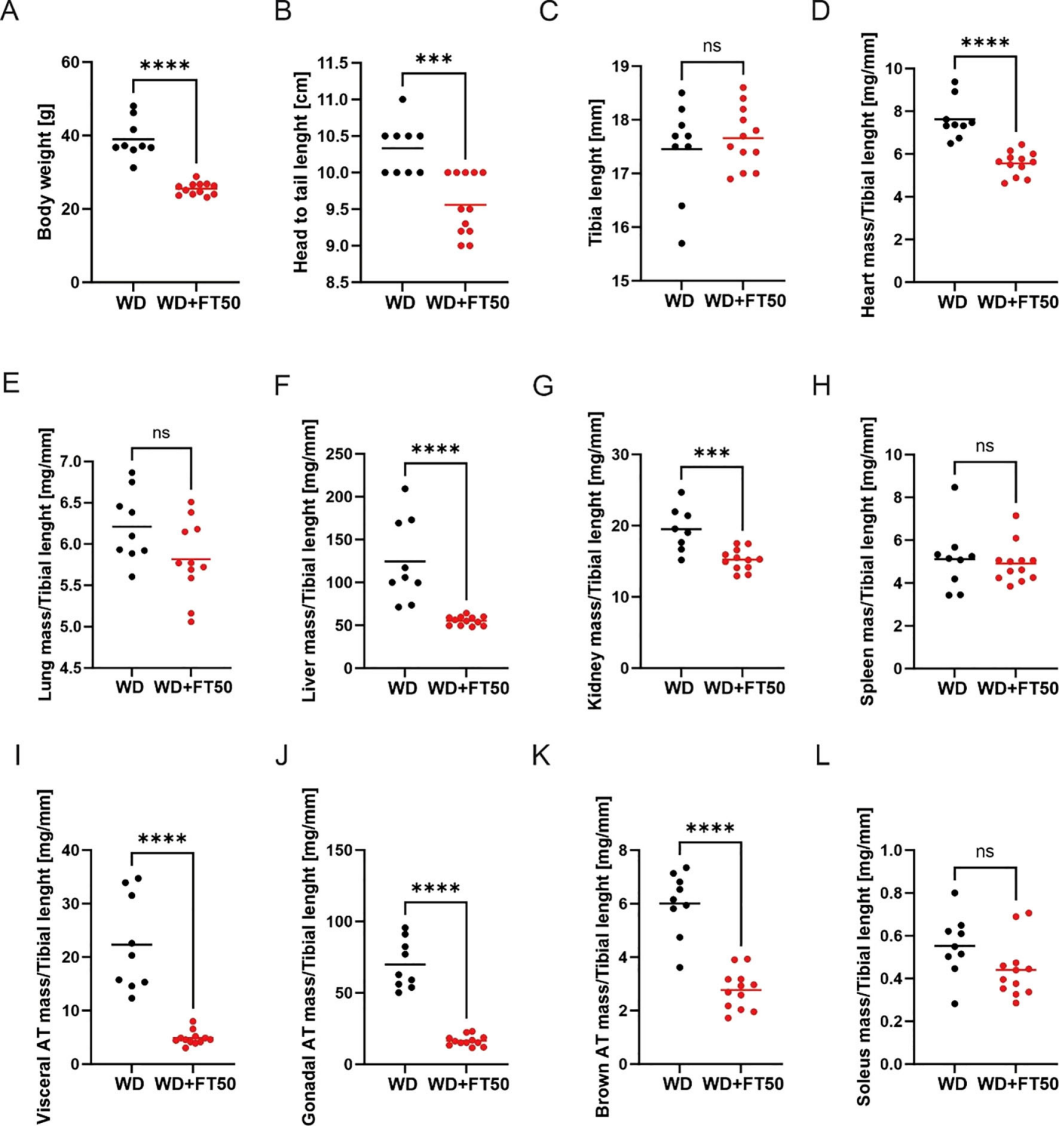
FT50 supplementation positively affects organ health and fat distribution. **(A)** Body weight was higher in WD (39.0 g, n=9) compared to WD+FT50 (25.5 g, n=12, ****p<0.0001). **(B)** Head-to-tail length was higher in WD (10.33 cm, n=9) compared to WD+FT50 (9.56 cm, n=12, ***p=0.0003). **(C)** Tibia length did not differ between WD (17.5 mm, n=9) and WD+FT50 (17.7 mm, n=12, ns). **(D)** Heart mass/tibia length was higher in WD (7.63 mg/mm, n=9) compared to WD+FT50 (5.56 mg/mm, n=12, ****p<0.0001). **(E)** Liver mass/tibia length was higher in WD (124.4 mg/mm, n=9) compared to WD+FT50 (55.3 mg/mm, n=12, ****p<0.0001). **(F)** Kidney mass/tibia length was higher in WD (19.5 mg/mm, n=8) compared to WD+FT50 (15.2 mg/mm, n=12, ***p=0.0006). **(G)** Lung mass/tibia length showed a tendency to be higher in WD (6.21 mg/mm, n=9) vs. WD+FT50 (5.82 mg/mm, n=11, p=0.0650). **(H)** Spleen mass/tibia length did not differ between WD (5.11 mg/mm, n=9) and WD+FT50 (4.92 mg/mm, n=12, ns). **(I)** Visceral AT mass/tibia length was higher in WD (22.3 mg/mm, n=9) compared to WD+FT50 (4.85 mg/mm, n=12, ****p<0.0001). **(J)** Gonadal AT mass/tibia length was higher in WD (69.8 mg/mm, n=9) compared to WD+FT50 (16.5 mg/mm, n=12, ****p<0.0001). **(K)** Brown AT mass/tibia length was higher in WD (6.01 mg/mm, n=9) compared to WD+FT50 (2.77 mg/mm, n=12, ****p<0.0001). **(L)** Soleus muscle mass/tibia length showed a tendency to be higher in WD (0.55 mg/mm, n=9) vs. WD+FT50 (0.44 mg/mm, n=12, p=0.081). Unpaired t-tests. ns, non significant.

**Figure 7:**
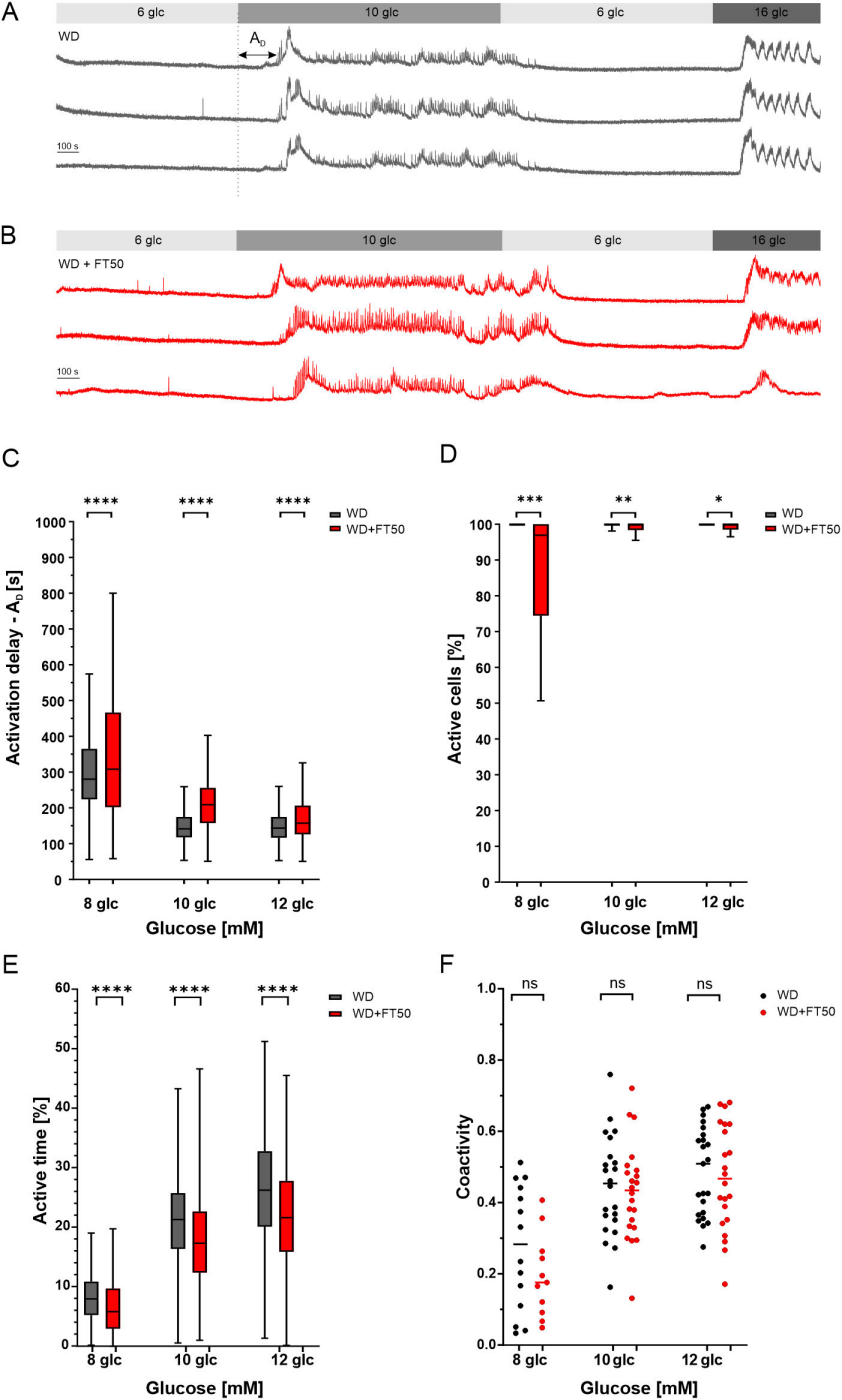
Effect of FT50 on beta cell activity in response to glucose. **(A)** Representative traces of [Ca^2+^]_IC_ from beta cells in WD group. **(B)** Representative traces of [Ca^2+^]_IC_ from beta cells in WD+FT50 group. **(C)** Median activation delay of cytosolic calcium increase ([Ca^2+^]_IC_) in response to stepwise glucose stimulation to 8, 10, or 12 mM in islets from WD and WD+FT50 groups. FT50 significantly prolonged activation delays at all glucose concentrations compared with WD alone (8 mM: median 280.3 s vs. 308.2 s, n = 1278 vs. 818 cells, p < 0.0001; 10 mM: median 141.4 s vs. 209.0 s, n = 1733 vs. 1788 cells, p < 0.0001; 12 mM: median 143.4 s vs. 157.3 s, n = 1975 vs. 2094 cells, p < 0.0001, ****). **(D)** Distribution of the percentage of active cells within islets reveals glucose-dependent heterogeneity. FT50 preserved physiological heterogeneity at low glucose, whereas WD islets displayed premature recruitment of beta cells. Significant differences were observed at 8 mM (median 100.0 vs. 96.9, n = 18 vs. 17 islets, p = 0.0008, ***), 10 mM (median 100.0 vs. 100.0, n = 22 vs. 29 islets, p = 0.0048, **), and 12 mM (median 100.0 vs. 100.0, n = 26 vs. 26 islets, p = 0.0164, *). Mann–Whitney U tests. **(E)** Active time of individual beta cells during the plateau phase at 8, 10, and 12 mM glucose. FT50 significantly shortened the active time at all tested glucose concentrations compared with WD alone (8 mM: median 8.0 s vs. 5.8 s, n = 799 vs. 648 cells, p < 0.0001; 10 mM: median 21.3 s vs. 17.3 s, n = 1711 vs. 1188 cells, p < 0.0001; 12 mM: median 26.2 s vs. 21.6 s, n = 1896 vs. 1378 cells, p < 0.0001, ****). Mann–Whitney U tests. **(F)** Coactivity of beta cells within individual islets at 8, 10, and 12 mM glucose. FT50 did not significantly alter coactivity at any glucose concentration (8 mM: mean 0.27 vs. 0.19, n = 14 vs. 11 islets, p = 0.20; 10 mM: mean 0.34 vs. 0.30, n = 15 vs. 12 islets, p = 0.39 (ns); and 12 mM: mean 0.41 vs. 0.37, n = 16 vs. 13 islets, p = 0.32 (ns)), indicating that coordinated beta cell activity was preserved. Unpaired t tests. Means ± SD are presented.

Following the sacrifice, tissues were harvested for subsequent analysis. **Figure 6A** illustrates that animals on a WD supplemented with FT50 had significantly lower body weights at the time of sacrifice compared to those on a WD alone. Similarly, **Figure 6B** shows that the FT50-supplemented group had a significantly shorter body length, measured from the tip of the snout to the base of the tail. However, **Figure 7C** indicates no significant differences in tibia length between the two groups. The lack of significant differences in tibia length between the two groups suggests that FT50 does not affect bone growth, which is important for ensuring that the observed changes in organ weights and body measurements are not due to altered skeletal development.

**Figures 6D–L** presents the weights of individual organs at the time of sacrifice for both groups. In human studies, organ measurements are typically normalized to account for size differences, a process known as indexing, which is often done using body weight, body surface area, or body mass index. In laboratory rodents, particularly mice, indexing to tibia length is preferred to avoid bias from disease-related changes in body weight. Therefore, **Figures 6D–L** shows the weights of individual organs normalized to tibia length.

Statistically significant decreases were observed in the weights of the heart, liver, and kidneys, as well as in the amounts of gonadal, visceral, and brown fat depots, between the group of animals receiving WD alone and those receiving WD supplemented with FT50. In contrast, there were no significant differences in the weights of the spleen, lungs, and soleus muscle. This indicates that FT50 supplementation might positively affect organ health and fat distribution.

### 3.7 The effect of FT50 on glucose-dependent calcium activity in pancreatic beta cells

To evaluate the impact of FT50 on beta cell function into more detail, we investigated the glucose-dependent calcium activity in the islets of Langerhans. All mice used in the study contributed to the calcium imaging datasets. We placed individual acute pancreas tissue slices into a recording chamber and perifused them with varying stimulatory glucose concentrations using a square pulse-like protocol (6 – 8–6 mM, 6 – 10–6 mM, or 6 – 12–6 mM). Each islet of Langerhans was subjected to a single protocol. Across slices obtained from each animal, all three stimulation protocols were performed. When glucose concentration was elevated, pancreatic beta cells exhibited a characteristic response: a transient rise in cytosolic calcium concentration followed by a sustained plateau with superimposed high-frequency oscillations. Representative traces for both groups are shown in **Figures 7A, B**. As expected, beta cells displayed a glucose-dependent activation delay (A_D_) —the temporal lag between stimulations and response ([Ca^2+^]_IC_ increase) - which progressively shortened with higher glucose concentrations. Activation delays were calculated for individual beta cells. In our experiments, the median activation delay at 8 mM glucose was 280.3 s in the WD group (n= x from y islets) and 308.2 s in the WD+FT50 group when stimulating the islets with 8 mM glucose (**Figure 7C**). Increasing glucose to 12 mM reduced the delay by 50% in both groups. Importantly, across all tested glucose concentrations, activation delays were significantly longer in the WD+FT50 group compared to the WD group. Furthermore, considerable cell-to-cell heterogeneity in activation delays was observed, and this variability was glucose-dependent (**Figure 7D**). In healthy islets, such heterogeneity at low glucose concentrations is a normal physiological feature, with full beta cell recruitment occurring only at glucose levels above 10 mM. Our results show that in the WD group, beta cells respond already at lower glucose concentrations, consistent with premature activation and potential hypersecretion. In contrast, beta cells in the WD+FT50 group retained physiological heterogeneity at low glucose, resembling the response pattern of healthy islets. Statistical analysis confirmed that FT50 significantly prolonged the glucose-induced activation delay relative to WD alone (p < 0.0001). This suggests that WD islets may exhibit insulin hypersecretion, a phenomenon commonly observed in the early stages of type 2 diabetes, whereas the addition of FT50 preserves a more physiological response profile (60, 61).

We next examined beta cell activity during the plateau phase by quantifying the proportion of time cells remained active (active time, AT) and the degree of coordinated activity between cells (coactivity) at different glucose concentrations. Active time was calculated for individual beta cells, whereas coactivity was quantified at the level of the islet. As shown in **Figure 7E**, AT increased with glucose in both groups, but was consistently higher in WD islets compared with WD+FT50 cells. This difference was highly significant at 8, 10, and 12 mM glucose (p < 0.0001 for all comparisons). In contrast, coactivity was not significantly different between WD and WD+FT50 islets across tested glucose concentrations (**Figure 7F**), indicating that FT50 did not affect the synchronization of beta cell responses.

These findings indicate that FT50 elicits beta cells responses with a longer temporal delay, recruits fewer beta cells at any given glucose concentration, decreases active time during the plateau phase, while preserving coordinated beta cell activity within the islet.

## 4 Discussion

### 4.1 FT50 and body weight regulation

Twelve weeks of WD feeding in C57BL/6J mice resulted in a more than 50% increase in body weight, consistent with previous reports that WD accelerates the onset of MetS and T2DM (19, 44, 45). Unlike most experimental models that rely on high-fat diets, approaches that only partially mimic human diabetes pathophysiology, WD-induced MetS and T2DM are considered more physiologically relevant, enhancing the translational value of this model (19, 62).

Remarkably, in animals receiving WD supplemented with FT50, body weight remained stable throughout the 12-week period, with an overall reduction of approximately ~4%, similar to what was observed in mice fed a HFD treated with dietary tannic acid (63). In our study, mice not only avoided excessive weight gain but also exhibited significantly lower fasting and non-fasting blood glucose levels, despite unchanged food intake. This observation suggests that FT50 exerts its effects through mechanisms other than appetite suppression. One plausible mechanism, supported by previous studies, involves reduced intestinal nutrient absorption or storage (64–68). Mechanistically, ellagitannins are known to reduce macronutrient digestibility by forming complexes with proteins, lipids, and carbohydrates, thereby limiting substrate availability for storage (31–33, 36, 69–71). This is consistent with our observation that FT50-fed mice maintained a stable body weight and exhibited markedly lower adiposity, including visceral, gonadal, and brown fat depots. Importantly, tibia length did not differ between groups, excluding growth retardation as a confounder. Beyond its effects on adiposity, FT50 significantly reduced liver, heart, and kidney weights, suggesting protection against WD-induced organ hypertrophy and lipid accumulation, particularly hepatic steatosis — a major driver of systemic insulin resistance. These changes may result from both decreased lipid absorption, potentially via pancreatic lipase inhibition, and improved hepatic lipid metabolism (69–72). The observed organ-specific benefits underscore the need for deeper mechanistic exploration in the future, such as lipidomic and transcriptomic profiling, to clarify whether FT50 modulates lipid handling at both intestinal and hepatic levels.

Another noteworthy observation was the substantial increase in water intake among mice receiving tannins. Although the underlying mechanism remains unclear, our preliminary (unpublished, not shown) data suggest that this effect may be linked to reduced intestinal water absorption, resulting in higher water content in the feces. Importantly, despite this shift, the animals did not develop diarrhea or soft stools, indicating that the effect is subtle rather than pathological. The increased fecal water loss could therefore explain the compensatory rise in water intake observed in the FT50 group.

If tannins inhibit intestinal glucose absorption, a concurrent reduction in water reabsorption is expected, as water transport is closely coupled to glucose uptake via Na^+^/glucose cotransporters (73). Impaired glucose transport would therefore decrease osmotic water movement, increase fecal water content, and drive compensatory polydipsia. This observation supports the hypothesis that FT50 modulates gastrointestinal nutrient and fluid handling, although confirmation requires metabolic cage studies.

### 4.2 Improvement in glucose metabolism and insulin sensitivity

As expected, prolonged WD feeding induced progressive deterioration in glucose homeostasis, as evidenced by a significant increase in AUC during ipGTT after 8 and 12 weeks. This finding is consistent with previous reports showing that WD accelerates the onset of MetS and T2DM by promoting obesity, systemic inflammation, and insulin resistance (62, 74, 75). In contrast, mice receiving WD supplemented with FT50 exhibited a strikingly different metabolic profile. Not only was the development of glucose intolerance completely prevented, but glucose sensitivity improved significantly as early as week 4 and remained enhanced throughout the 12-week intervention. Similarly, ipITT revealed that WD-fed mice developed impaired glucose handling in response to insulin early in the study, as indicated by increased AUC and a less negative K_ITT_ slope. These changes were evident in week 4 and became more pronounced at weeks 8 and 12. Conversely, FT50 supplementation attenuated these changes, with AUC and K_ITT_ values remaining stable and significantly different from those of WD-fed controls. Given the modified ITT protocol, these results should be interpreted as supportive of altered insulin action rather than a direct measure of insulin sensitivity. Given the modified ITT protocol, these findings should be interpreted as reflecting an integrated *in vivo* glucose-lowering response rather than a direct measure of insulin sensitivity. This was further supported by significantly lower HOMA-IR and higher %S values in animals supplemented by FT50, consistent with improved markers of insulin sensitivity. Importantly, fasting glucose and insulin levels, together with HOMA-IR, represent indirect estimates of insulin sensitivity and should be interpreted with this limitation in mind. Accordingly, these findings indicate improvements in indirect markers of insulin-related physiology rather than direct measures of insulin action. This suggests that FT50 exerts a robust protective effect on metabolic regulation, preventing the cascade of metabolic disturbances typically associated with chronic WD intake. The mechanisms underlying these effects are likely multifactorial. Ellagitannins and their metabolites may modulate glucose metabolism through several potential pathways, including the inhibition of intestinal glucose absorption (76, 77), enhancement of insulin receptor signaling, and reduction of oxidative stress and inflammation (78, 79). However, these mechanisms remain hypothetical and were not directly assessed in the present study. The early and sustained improvement in glucose tolerance observed in the FT50 group suggests that intestinal mechanisms—such as reduced carbohydrate digestibility and absorption—may play a primary role, potentially complemented by systemic effects on insulin related physiology.

### 4.3 Effects on insulin secretion and beta cell function

Our findings demonstrate that FT50 supplementation not only improves glucose tolerance and is associated with improved markers of insulin sensitivity but also modulates the dynamics of insulin secretion in WD-fed mice. Progressive hyperinsulinemia was observed over time in the WD group, reflecting a compensatory response to worsening insulin resistance. This pattern is consistent with the pathophysiology of early T2DM, in which beta cells increase insulin output to maintain normoglycemia (80–84).

In contrast, FT50-treated mice exhibited significantly lower fasting and early-phase insulin levels at week 8, with this effect becoming more pronounced at week 12. These changes occurred despite similar glucose challenges, suggesting that FT50 is associated with improved systemic insulin responsiveness, thereby reducing the need for compensatory hyperinsulinemia. This interpretation is supported by HOMA-IR and %S calculations, which confirmed that FT50 prevented the rise in insulin resistance observed in WD-fed controls and is consistent with preserved or improved markers of insulin sensitivity throughout the study.

Interestingly, whole-animal assessments of beta cell function did not reveal significant differences between groups. Although a modest trend toward lower values was observed in FT50-treated mice at week 12, this likely reflects reduced secretory demand rather than impaired beta cell capacity, consistent with the markedly improved glucose tolerance in these animals. This distinction is crucial, as chronic beta cell overstimulation—rather than reduced function—drives beta cell exhaustion and accelerates T2DM progression (85–88).

However, the absence of differences at the systemic level should not be interpreted as a lack of beta cell effects. Because beta cells are highly heterogeneous, whole-islet or whole-animal measurements can mask cell-specific alterations. To uncover these, we performed *ex vivo* Ca^2+^ imaging in individual beta cells within pancreas tissue slices. Here, clear functional differences emerged. In WD-fed mice, beta cells exhibited shortened activation delays and premature recruitment at lower glucose concentrations, consistent with heightened excitability and a hypersecretory phenotype. In contrast, FT50 prolonged activation delays across all glucose steps and preserved physiological heterogeneity at low glucose – more closely resembling the response pattern of healthy beta cells (60, 61). This suggests that FT50 normalizes the glucose activation threshold and prevents inappropriate beta cell activation under mild glycemic stimuli.

During the plateau phase, FT50 significantly shortened the active time of individual beta cells without altering coactivity, indicating that FT50 reduces the duration of Ca²-dependent secretory activity while maintaining coordinated islet network behavior (89). Functionally, this pattern is consistent with a lower secretory burden and reduced intracellular Ca^2+^ load, which may contribute to protection against ER stress and oxidative damage, key drivers of beta cell dysfunction under metabolic stress (90, 91).

### 4.4 Study limitations and future directions

Several limitations of the present study should be acknowledged. First, the mechanisms underlying the metabolic effects of FT50 remain to be fully elucidated. Based on the known biochemical properties of ellagitannins, potential mechanisms may include modulation of intestinal nutrient absorption and intestinal water handling. Preliminary analyses indicated increased fecal water content in FT50-treated mice as well as changes in the expression of selected intestinal glucose transporters; however, these data are part of a separate investigation focused on intestinal mechanisms and are therefore not included in the present manuscript. No measurements of fecal energy content, urine output, or metabolic cage parameters were performed in this study. Second, conclusions regarding beta cell protection are limited to functional measurements obtained from calcium imaging in pancreatic tissue slices. Structural parameters, such as beta cell mass, markers of cellular stress, or long-term preservation of islet architecture, were not evaluated in this study. Third, assessment of insulin-related physiology was based on *in vivo* tests and derived indices that reflect integrated systemic responses. While fasting glucose and insulin levels, HOMA-IR, glucose tolerance, and insulin tolerance tests are widely used in this context, they represent indirect measures of insulin sensitivity and insulin action. In particular, the modified ITT protocol used in this study does not isolate insulin-specific effects, as glucose dynamics are influenced by multiple physiological processes, including endogenous insulin secretion, hepatic glucose output, and neuroendocrine regulation. However, these approaches are commonly used to characterize whole-body metabolic responses *in vivo*, and in the present study they were interpreted accordingly, with emphasis on relative differences between experimental groups. Fourth, only male mice were used, as male C57BL/6J mice are more susceptible to the development of metabolic syndrome and type 2 diabetes in the Western Diet model, whereas disease progression in females is typically slower or less pronounced (92–94). Nevertheless, sex differences in metabolic disease and beta cell responses are well documented, and future studies should therefore include both sexes. In addition, the twelve-week dietary intervention represents an early stage of diet-induced metabolic dysfunction in this model, and later stages of disease progression and beta cell decompensation were not examined. Finally, independent replication and further mechanistic studies will be required to confirm these findings and to better define the translational potential of chestnut-derived ellagitannins.

## 5 Conclusion

In conclusion, FT50 supplementation attenuated several features of early diet-induced metabolic dysfunction in WD-fed C57BL/6J male mice, including weight gain, glucose intolerance, changes consistent with impaired insulin action, hyperinsulinemia, hypertriglyceridemia, and excess fat accumulation. Importantly, FT50 achieved these protective effects without reducing caloric intake, suggesting that tannin supplementation could complement, rather than replace, dietary and lifestyle interventions in humans. However, translation of dosing regimens from mice to humans requires careful consideration and clinical evaluation, particularly given the potential gastrointestinal side effects of high-tannin intake and the fact that the bioavailability and metabolism of ellagitannins differ between species. Taken together, these findings suggest that chestnut extract represents a promising bioactive dietary intervention for modulating early metabolic alterations, while emphasizing the need for further mechanistic studies, independent replication, and evaluation in human populations.

## Data Availability

The raw data supporting the conclusions of this article will be made available by the authors, without undue reservation.

## Supplementary material

Supplementary Figure 1 FT50 increases the water consumption Food intake in both groups was comparable **(A)**, while water intake increased in WD+FT50 during the diet, reaching almost 3 times the WD group **(B)**. Due to small sample size (2 in WD and 3 in WD+FT50) data from A and B were not statistically validated.
