# Supplementary figures and images for: Chestnut-derived ellagitannins (FT50) protect against western diet-induced metabolic dysfunction and preserve beta cell function in mice
Source: Front Endocrinol (Lausanne). 2026 Apr 30;17:1802808. doi: 10.3389/fendo.2026.1802808 (PMC13173460; doi:10.3389/fendo.2026.1802808)

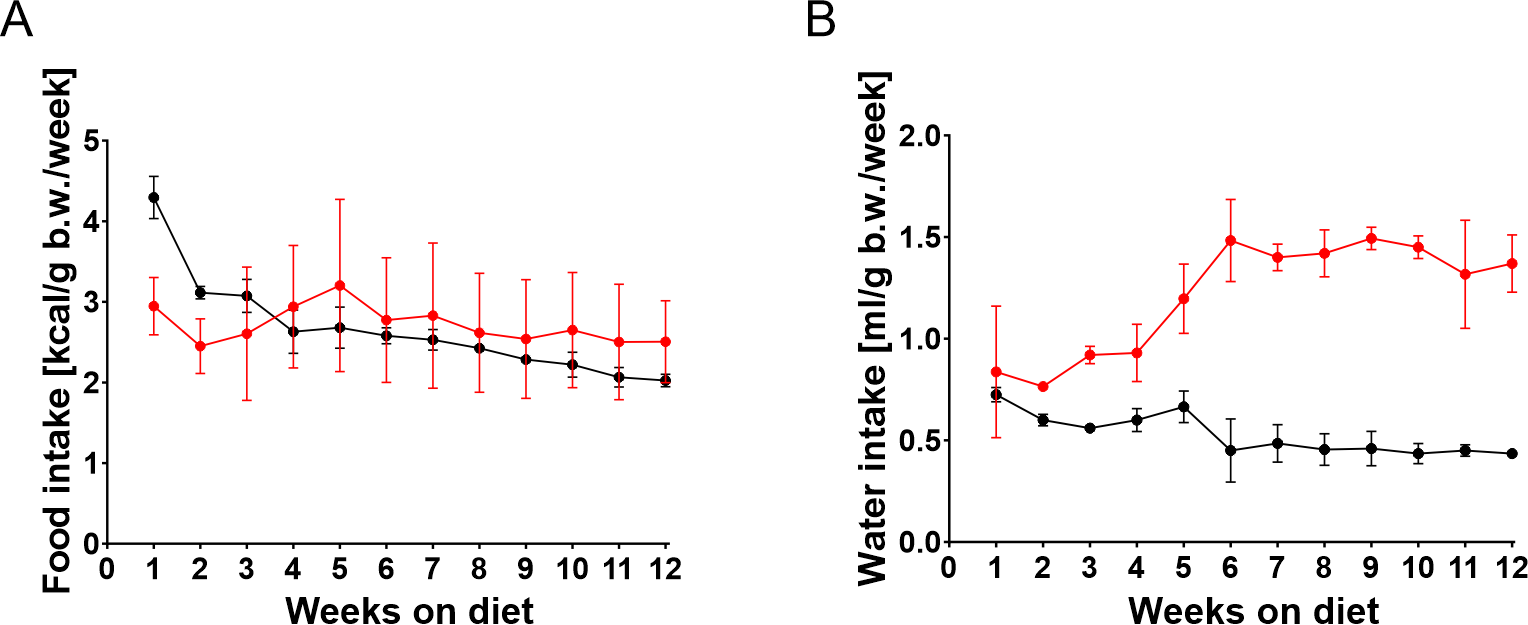

Supplement: Supplementary Figure 1 — FT50 increases the water consumption Food intake in both groups was comparable (A), while water intake increased in WD+FT50 during the diet, reaching almost 3 times the WD group (B). Due to small sample size (2 in WD and 3 in WD+FT50) data from A and B were not statistically validated. [file Image1.tif]
