# Supplementary material for: Chestnut-derived ellagitannins (FT50) protect against western diet-induced metabolic dysfunction and preserve beta cell function in mice
Source: Front Endocrinol (Lausanne). 2026 Apr 30;17:1802808. doi: 10.3389/fendo.2026.1802808 (PMC13173460; doi:10.3389/fendo.2026.1802808)
Supplement: Supplementary file 2 [file Table1.pdf]

Supplement Table 1: The composition and caloric value of the Western diet (WD) and the Western diet with chestnut extract (WD+FT50)

	**Westn Diet - D12079B (WD)**		**Western Diet with FT50 (WD+FT50)**	
	gm%	*kcal%*	gm%	*kcal%*
Proteins	*19.8*	*17*	*19.2*	*17*
Carbohydrates	*50.0*	*43*	*48.5*	*43*
Fats	*21.0*	*40*	*20.3*	*40*
Total		*100*		*100*
kcal/gm	*4.68*		*4.54*	
Ingredient	gm	*kcal*	gm	*kcal*
Casein	195	*780*	195	*780*
DL-Methionine	3	*12*	3	*12*
Maize starch	50	*200*	50	*200*
Maltodextrin 10	100	*400*	100	*400*
Sucrose	341	*1364*	341	*1364*
Cellulose	50	*0*	50	*0*
Milk fats, anhydrous	200	*1800*	200	*1800*
Corn oil	10	*90*	10	*90*
Mineral Mix S10001	35	*0*	35	*0*
Calcium carbonate	4	*0*	4	*0*
Vitamin Mix V10001	10	*40*	10	*40*
Cholin bitartrate	2	*0*	2	*0*
Cholesterol, USP	1.5	*0*	1.5	*0*
Ethoxyquin	0.04	*0*	0.04	*0*
Chestnut extract	0	*0*	*31*	*0*
FD&C Red Dye #40	0	*0*	0.05	*0*
Total	*1001.54*	4686	*1032.59*	4686
Chestnut extract (g/kg)	*0*		*30.0*	
